# Anti-inflammatory and burn injury wound healing properties of the shell of *Haliotis diversicolor*

**DOI:** 10.1186/s12906-016-1473-6

**Published:** 2016-11-28

**Authors:** Zhi-Cheng Chen, Shing-Yi Sean Wu, Wei-Yang Su, Yuan-Chuan Lin, Yi-Hsin Lee, Wei-Hao Wu, Chun-Hong Chen, Zhi-Hong Wen

**Affiliations:** 1 Department of Marine Biotechnology and Resources, National Sun Yat-sen University, Kaohsiung, Taiwan; 2 Department of Chinese Medicine for Post-Baccalaureate, I-Shou University, Kaohsiung, Taiwan; 3 Graduate Institute of Basic Medical Science, China Medical University, Taichung, Taiwan; 4 Doctoral Degree Program in Marine Biotechnology, National Sun Yat-Sen University, Kaohsiung, Taiwan; 5 Doctoral Degree Program in Marine Biotechnology, Academia Sinica, Taipei, Taiwan

**Keywords:** *Haliotis diversicolor*, Burn injury, Wound healing, Transforming growth factor-beta

## Abstract

**Background:**

The shell of *Haliotis diversicolor*, or shijueming (SJM), is a type of traditional Chinese medicine. The SJM has appeared in historical records as early as the third and fourth centuries. Historical records have revealed that SJM had mainly been used to treat eye diseases. After the Qing Dynasty (1757), records had emerged, detailing the use of SJM for treating skin injuries, particularly for treating poorly managed ulcers or traumatic wounds. Furthermore, in our anti-inflammation-screening system, SJM significantly inhibited the expression of pro-inflammatory proteins. Previous studies have yet to adopt an animal model to verify the phenomenon and described in the historical records regarding the efficacy of SJM in promoting wound healing. Besides, the mechanism of wound healing effect of SJM is also not clear.

**Methods:**

This study applied in vitro and in vivo models, tissue section analysis, and western blotting to evaluate the effect of SJM on wound healing. The RAW 264.7 cells were used in anti-inflammatory activity assay and phagocytic assay. Male Wistar rats were used to evaluate the effect of SJM on burn injury healing. A copper block (2 × 2 cm, 150 g) preheated to 165 °C in a dry bath was used to contact the skin area for 10 s, thus creating a full-thickness burn injury. The results were analyzed by hematoxylin and eosin staining, picrosirius red staining and Western blotting.

**Results:**

The results revealed that in the in vitro model, the presence of SJM decreased the inducible nitric oxide synthase (iNOS) expression and enhanced the functions of macrophages. The results of the rat burn injury model revealed that SJM decreased neutrophil infiltration, promoted wound healing, thus increasing the collagen I content and promoting the expression of transforming growth factor-beta 1 (TGF**-**β1) protein. We speculate that the effect and mechanism of SJM on promoting wound healing is related to macrophage activation. In the inflammation phase, SJM alleviates inflammation by inhibiting iNOS expression and removing neutrophils through phagocytosis. Furthermore, SJM induces the secretion of TGF-β1, converting collagen during the tissue remodeling phase.

**Conclusions:**

According to our review of relevant literature, this is the first study that applied an evidence-based method to verify that SJM alleviates inflammation, enhances phagocytosis, and triggers wound healing after burn injury. The study findings reveal that SJM provides a promising therapeutic option for treating burn injury.

## Background

Burn injuries, particularly those at the full-thickness level, can develop into acute wounds that are difficult to manage and heal [1]. Currently, standard treatment for burn injuries involves using silver-containing antibiotics to prevent infection [2]. Because silver ion is highly reactive, it can bond with deoxyribonucleic acid, ribonucleic acid, chloride ions, and other negatively charged proteins [3]. Additionally, medical dressings are used to maintain the hydration environment and air permeability of a burn injury [4]. However, the aforementioned treatment methods cannot be used to alleviate inflammation or facilitate healing a burn injury. Previous studies have indicated that silver sulfadiazine is toxic to keratinocytes and fibroblasts, thus decreasing the healing rate of a wound [5–8]. Consequently, scholars have suggested that the use of silver-containing drugs should be avoided when treating a wound [3]. Therefore, developing safe drugs that promote wound healing is necessary.

Early studies have verified that macrophages facilitate healing wounds through promoting angiogenesis and collagen formation [9–12]. Laboratory rats with macrophage depletion exhibit reductions in wound healing rate, collagen formation, granulation tissue, angiogenesis, and secretion of transforming growth factor-beta (TGF-β1; [13–15]), indicating that macrophages facilitate healing wounds.

The shell of *Haliotis diversicolor*, or shijueming (SJM), is a type of traditional Chinese medicine. The exact time period in which this medicine was first used is debatable, but this medicine has appeared in historical records as early as the third and fourth centuries. Historical records have revealed that SJM had mainly been used to treat eye diseases. After the Qing Dynasty (1757), records had emerged, detailing the use of SJM for treating skin injuries, particularly for treating poorly managed ulcers or traumatic wounds. In recent years, multiple scholars have analyzed the pharmaceutical property of SJM using scientific methods and techniques, thereby providing systematic, scientized evidence supporting the use of this medicine. Accordingly, the medical functions of SJM can be further explored and verified.

Several studies have demonstrated the use of lipopolysaccharide (LPS) challenged RAW 264.7 murine macrophage cells, as a well-established model for in vitro anti-inflammatory screening. Using this screening model, we have successfully illustrate the several marine-derived substances can produce efficacious anti-inflammatory activity in in vitro and in vivo models [16–19]. Thus, screening for inhibitors of the pro-inflammatory mediators in marine natural bioactive substances is a promising avenue for drug development. Inflammation is the initial phase of wound healing which the macrophages mainly take the role. Resident macrophages are activated and generate inflammatory mediators such as nitric oxide (NO) to prevent infections caused by the invasions of foreign pathogens. However, prolonging the inflammatory response and delaying granulation will cause delayed healing. Because excessive production of NO also damages normal tissue [20, 21]. Thus, the inhibition of inflammation is essential to wound treatment. In our anti-inflammation-screening system, SJM significantly inhibited the expression of pro-inflammatory proteins. Moreover, decrease in oxidative stress may lead to acceleration of the wound healing [22, 23]. A recent study reported that a SJM solution significantly increased the survival rate of human lens epithelial cells that are damaged by hydrogen peroxide-induced oxidative stress [24]. Therefore, based in the effect of protect oxidative damage and anti-inflammation potential, SJM may has potential to enhance wound healing. This study applied in vitro and in vivo models, tissue section analysis, and western blotting to evaluate the effect of SJM on wound healing.

## Methods

### *Haliotis diversicolor* shell preparation

**Fig. 1:**
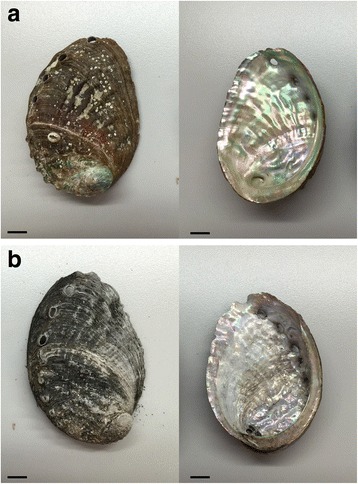
The SJM before (**a**) and after (**b**) calcined. SJM was placed in a metal pot and calcined, note the surface gloss was altered after calcined. Scale = 1 cm

The shell of *Haliotis diversicolor* (Zheng-Der Chinese herbal apothecary, Taipei, Taiwan) was placed in a metal pot and calcined (300 °C for 15 min) until its surface gloss was altered (Fig. 1). The shell was then cooled and ground to fine powder (particle diameter < 75 μm). Because SJM is slightly soluble, the SJM powder was dissolved in a culture medium, and only the supernatant of the medium was used in the in vitro experiment. Specifically, the SJM powder was weighed and then loaded into Dulbecco’s modified Eagle’s medium (DMEM, Invitrogen, Grand Island, NY, USA) containing 10% fetal bovine serum. The resulting medium was evenly mixed, sonicated for 15 min, and centrifuged for 10 min at 180 × g. The supernatant was acquired and loaded into a new centrifuge tube and then centrifuged for 10 min at 400 × g. The resulting supernatant was filtered using 0.22 μm membrane to obtain a nonbacterial SJM solution. The solution was kept in a 4 °C refrigerator and used as the culture medium. In this study, when 1 g of SJM powder was added to 10 ml of cell growth medium, the resulting supernatant solution was defined as having a concentration of 10 mg/ml. Regarding the animal experiment, the SJM powder was added to a mineral oil (Macron, Center Valley, PA, USA) to prepare a high-concentration 600 mg/mL solution (SJM-H) and a low-concentration 200 mg/mL solution (SJM-L).

### Cell culture and cell survival assay

The RAW 264.7 cells were grown in 1:1 DMEM (Invitrogen, Grand Island, NY, USA) containing 10% heat-inactivated fetal bovine serum, penicillin G (100 U/ml), and streptomycin (100 μg/ml). For the cell survival assay, the cells were seeded in 96-well culture dishes (Corning, NY, USA) at an initial density of 3000 cells/well. The SJM at suitable doses was added to cell culture. The cells were cultured in DMEM medium within a 5% CO_2_ atmosphere humidified incubator at 37 °C for 24 h. After 24 h incubation, 10% AlamarBlue (Invitrogen, Grand Island, NY, USA) was aseptically added to measure cell viability. The vehicle control group (without SJM) was defined as 100%. The optical density (OD) of the supernatants were quantified at 570 nm, and the cell viabilities were analyzed according to the following formula: Cell viability (%) = (OD_SJM_ – OD_blank_)/(OD_control_ – OD_blank_) × 100%.

### Anti-inflammatory activity assay

10 × 10^6^ RAW264.7 cells were cultured in a 10 cm culture dish and administered with lipopolysaccharide (LPS, 0.01 μg/ml; Sigma, St. Louis, MO, USA). Specifically, 1, 2, and 5 mg/mL SJM solutions were added into the culture dish and followed by the addition of LPS. After 16 h of LPS challenge, the protein expression of inducible nitric oxide synthase (iNOS) was analyzed by Western blotting.

### Phagocytic assay

A phagocytosis assay kit (Cayman Chemical, Ann Arbor, MI, USA) was used to test the phagocytic capability of macrophages. RAW 264.7 was cultured in a 24-well plate. Specifically, 1, 2, and 5 mg/mL SJM solutions were separately loaded into the selected wells. For every 1 ml of culture medium, 100 μl of Latex Beads-Rabbit IgG-FITC solution was loaded. The plate was cultured at 37 °C, and the culture medium was replaced after 8 h. Finally, the cultured cells were placed under a microscope to observe the conditions of the fluorescently-labeled latex beads.

### Neutral red phagocytic assay

RAW 264.7 cells were cultured in a 96-well plate. An adherent culture method was applied, and the cells were treated with 1, 2, and 5 mg/ml SJM solutions for 24 h. The positive control group was loaded with a 0.5 μg/ml LPS, followed by 100 μl of 0.075% neutral red solution (Sigma, St. Louis, MO, USA) prepared with 1× phosphate buffer saline (PBS). The plate was cultured at 37 °C for 1 h. The supernatant was removed, and the cultured cells were washed with 1× PBS twice to remove residual neutral red molecules that were not engulfed. Next, 100 μl of lysate (1:1 alcohol to 0.01% acetic acid) was used to lyse the cultured cells at room temperature for 1 h. Finally, an enzyme immunoassay analyzer (Epoch, BioTek Instruments, Winooski, VT, USA) was used to measure the absorbance value of the cells at 540 nm [25].

### Burn injury creation and medical dressing

The animal experiment was approved by the Animal Care and Use Committee of National Sun Yat-sen University (Approval No. 10503) and the experiment complied with the Guiding Principles in the Care and Use of Animals of the American Physiology Society. Efforts were taken to minimize animal discomfort and the number of animals used for the experiment. Male Wistar rats (400–450 g) were anesthetized with 2.5% isofurane. The hair on the backs of the rats was shaved off. A rat burn injury model was modified from previous studies [26–28]. Specifically, the target skin area was tightly stretched to ensure a flat contact surface. A copper block (2 × 2 cm, 150 g) preheated to 165 °C in a dry bath was used to contact the skin area for 10 s, thus creating a full-thickness burn injury. During the process of burn injury creation, the copper block was lightly shaken while pressed onto the skin surface to create a complete, square-shape wound. No additional pressure was exerted on the copper block to ensure that each burn injury was created using the similar pressure. The injured rats were randomly assigned into the following groups:

Control: no burn injury or medicine was given.

Burn + untreated: burnt but no medicine was given.

Burn + vehicle: burnt and then smeared with 0.2 ml of mineral oil.

Burn + SJM-H: burnt and then treated with 0.2 ml of 600 mg/ml SJM solution daily.

Burn + SJM-L: burnt and then treated with 0.2 ml of 200 mg/ml SJM solution daily.

The identical dosages of medicine were applied to the rats during the same time each day. Before each dressing, the wound was cleaned with a sterilized saline solution to remove residual medicine and foreign matters. A sterilized cotton swab was used to dry the saline solution, and then the medicine was evenly smeared onto the wound. Every two days, the rats were anesthetized and then placed on a copy stand. The rats were photographed under identical conditions using a digital camera (Coolpix P6000, Nikon, Japan). SPOT software (Diagnostic Instruments, Inc., Sterling Heights, MI, USA) was used to examine the area of each skin wound.

### Histopathological analysis

For the histopathological examination, the rats were sacrificed after perfusion with PBS and 4% paraformaldehyde as described previously [29, 30], and the skin was removed and fixed in 4% paraformaldehyde for 3 days. The specimens were dehydrated in a graded series of alcohol by rotary tissue processor (Sakura Finetek, Japan) and embedded with paraffin, and 2-μm sections were prepared for hematoxylin and eosin staining to assess the general and pathological changes. The sections were examination by light microscopy (DM 6000B, Leica Inc. Wetzlar, Germany) with a microscope digital image output system (idea SPOT, Diagnostic Instruments Inc., Steriling Heights, MI, USA). Histological sections stained in picrosirius red (picrosirius red stain kit, Polysciences, Warrington, PA, USA) and analyzed under polarized light were used to analysis of the collagen deposition after burn injury. Collagen fibers were analyzed according to their color which collagen I and collagen III respectively appear red and green.

### Western blotting

After 16-h incubation, the cells were washed with ice-cold PBS, lysed in ice-cold lysis buffer (50 mM Tris, pH 7.5, 150 mM NaCl, 1% Triton X-100, 100 mg/ml phenylmethylsulphonyl fluoride, 1 mg/ml aprotinin), and then centrifuged at 20 000 × g for 30 min at 4 °C. The supernatant was decanted from the pellet and retained for Western blot. Protein concentrations were determined using the DC protein assay kit (Bio-Rad, Hercules, CA, USA). The animals were sacrificed at 7 days after injury, the skin was collected and washed with ice-cold PBS and homogenized in lysis buffer using a Polytron homogenizer (Precellys®24 tissue homogenizer; Bertin Technologies, Rockville, MD, USA) at 4 °C. Samples were then ultracentrifuged for 1 h at 4 °C, and the supernatant was measured using a DC protein assay kit (Bio-Rad, Hercules, CA, USA).

The samples then electrophoresed through a tricine SDS-polyacrylamide gel and transferred to a polyvinylidene difluoride membrane (PVDF membrane; Immobilon-P, Millipore, 0.45-μM pore size). After blocking for 1 h at room temperature with 5% non-fat milk in Tris-buffered saline (TTBS; 137 mM NaCl, 20 mM Tris–HCl, 0.1% Tween-20®, pH 7.4), the PVDF membrane was then incubated overnight at 4 °C with primary antibodies: rabbit polyclonal antibody against TGF-β1 (1:1000; C﻿at. Ab92486; Abcam, Cambridge, MA, USA). The immunoreactive bands were visualized by enhanced chemiluminescence (ECL kit; Millipore, Bedford, MA) and by the UVP BioChemi Imaging System; and relative densitometric quantification was performed using LabWorks 4.0 software (UVP, Upland, CA). Monoclonal antibodies against β-actin were used as the internal loading control. The quantification data were expressed as a ratio of the protein of interest to β**-**actin. The relative variations between the bands of the various treatment samples and of the control group were calculated using the same image.

### Statistical analysis

All data are presented as the mean ± SEM. For the immunoreactivity data, the intensity of each test band is expressed as the integrated optical density (IOD), calculated with respect to the average optical density of the corresponding control (LPS or control) band. For statistical analysis, all of the data were analyzed by a one-way analysis of variance (ANOVA), followed by the Duncan’s method for multiple comparisons (Systat Software, San Jose, CA, USA). We defined a significant difference as *p* < 0.05.

## Results

### Promotion of macrophage proliferation and phagocytosis

**Fig. 2:**
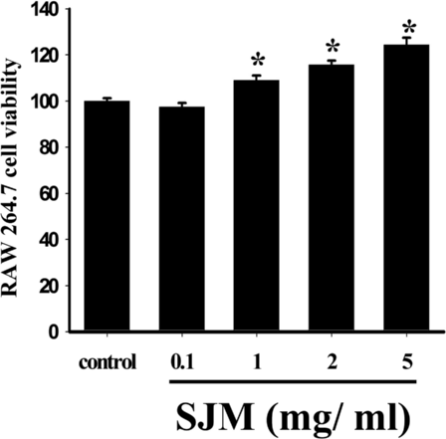
Effect of SJM on the survival rate of mice macrophage RAW 264.7. The survival rates of the groups treated with 1–5 mg/ml SJM increased significantly, indicating that SJM promotes macrophage proliferation. *denotes *p* < 0.05 compared with the control group

**Fig. 3:**
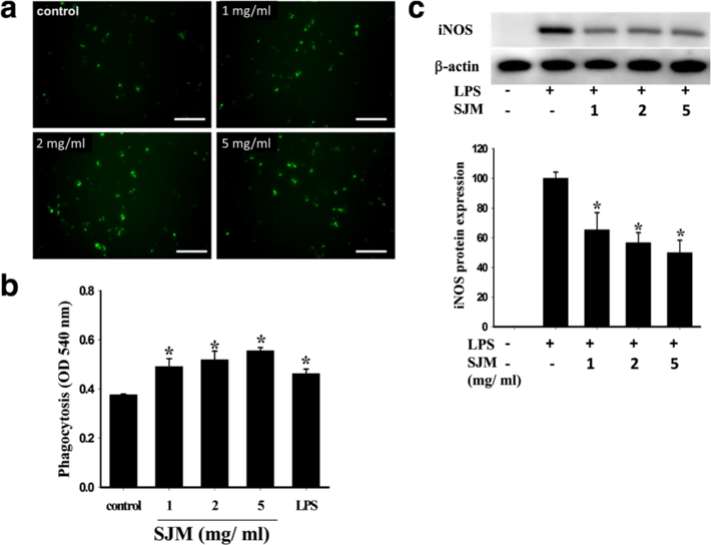
Effect of SJM on RAW 264.7 phagocytic capability and in vitro anti-inflammation. **a** The Cayman phagocytosis assay kit was applied to treat the cells with SJM for 8 h. Observing the cells under a microscope revealed that compared with the vehicle group, the SJM-treated cells exhibited enhanced ability in engulfing the fluorescent beads. **b** The neutral red experiment was conducted to observe the effect of SJM on the phagocytic capability of RAW 264.7. The statistical results showed that the absorbance values of the RAW 264.7 SJM- and LPS-treated cells were significantly higher than that of the control group, indicating that SJM enhances the phagocytic ability of macrophages. **﻿c﻿**The RAW 264.7 cells were treated with LPS and SJM for 16 h, and then western blotting was applied to detect the expression of iNOS in these cells. The results revealed that LSP induced RAW 264.7 to synthesize inflammation factor iNOS. However, SJM significantly inhibited LPS from inducing the synthesis of iNOS proteins. # denotes *p* < 0.05 compared with the control group and * denotes *p* < 0.05 compared with the LPS group; scale = 100 μm

The RAW 264.7 cells were treated with SJM solutions of various concentrations for 24 h. The survival rate of the control group was 100% (±1.2%). The survival rates of the cells treated with 0.1, 1, 2, and 5 mg/ml of SJM solutions were 96.7% (±1.2%), 108.8% (±2.1%), 113.5% (±1.3%) and 124.2% (±1.3%), respectively (Fig. 2). Particularly, the survival rates of the cells treated with 1–5 mg/ml SJM solutions were significantly higher than those of the control group, indicating that SJM promotes macrophage proliferation. Next, the effect of SJM on the phagocytic capability of macrophages was examined. Compared with the control group, the RAW 264.7 cells treated with SJM for 8 h exhibited enhanced capability in engulfing fluorescent beads (Fig. 3a). The neutral red phagocytic assay was also applied to determine the effect of SJM treatment on the phagocytic capability of macrophages. The results revealed that after treatment with 1, 2, and 5 mg/ml SJM solutions for 24 h, the macrophage cells exhibited absorbance values of 0.492 (±0.032), 0.520 (±0.035), and 0.556 (±0.013), respectively, at 540 nm. The control group and positive control group (treated with LPS) exhibited absorbance values of 0.377 (±0.002) and 0.463 (±0.018), respectively (Fig. 3b). The statistical analysis results showed that the absorbance values of the SJM treated group and positive control group were significantly higher than those of the control group. Therefore, SJM enhanced the phagocytic capability of macrophages.

### Anti-inflammation effect of SJM

Wound healing in the skin involves an inflammation stage to remove foreign microorganisms and dead cells. However, an extensive, chronic inflammation decreases the healing rate of a wound and even increases the probability of scarring. Therefore, this study applied LPS to induce macrophage inflammation and generate an inflammation factor, iNOS. The results revealed that compared with the control group, the cells treated with LSP exhibited an increased expression of iNOS, whereas the expression of iNOS was inhibited in those treated with 1, 2, and 5 mg/ml SJM (Fig. 3c).

### Effect of SJM on healing rate of burn injury

**Fig. 4:**
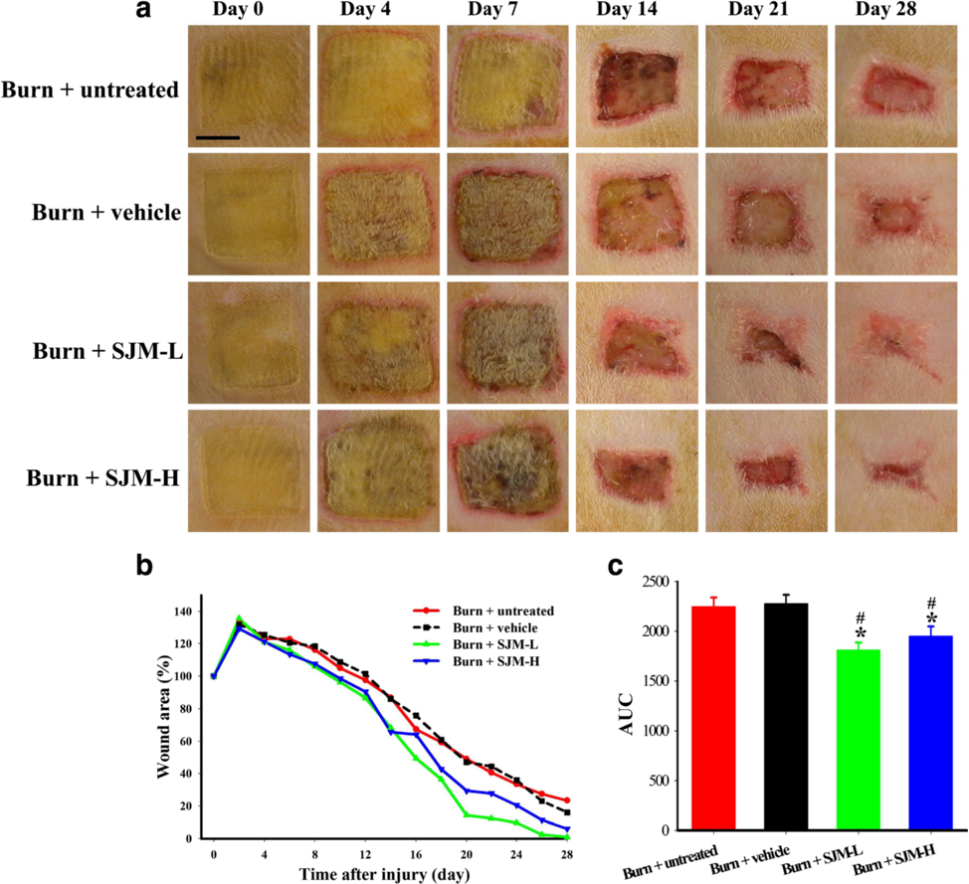
Effect of SJM on the burn injury appearances of the rats. The 165 °C copper block was used to create full-thickness burn injuries on the rats, and the wounds were photographed every two days. The wounds were continually observed for 28 days. (The images illustrate the wound appearances after 0, 4, 7, 14, 21, and 28 days. **b** The curves represent the wound area of each group (displayed as percentage value) versus number of days. The results revealed that the wound healing areas of the burn + vehicle and burn + untreated groups differed nonsignificantly, and the wound healing rates of burn + SJM groups were significantly higher than that of the burn + vehicle group. **c** The area under the wound area–time curve is shown for the burn + SJM-L and burn + SJM-H-treated groups, which exhibited significant improvement compared to the burn + vehicle group. Scale = 1 cm * denotes *p* < 0.05 compared with the burn + untreated group and # denotes *p* < 0.05 compared with the burn + vehicle group

Figure 4a illustrates the images of the burn injuries after 0, 4, 7, 14, 21, and 28 days. The results revealed that although the wound of each group gradually healed with time, the wounds of the burn + SJM groups had begun to heal noticeably after 14 days. After 28 days, the wound of the burn + SJM-L group was mostly healed. Calculating the wound areas of different groups (Fig. 4b) revealed that the wound healing rates of the vehicle and burn groups did not differ, whereas those of the burn + SJM-H and burn + SJM-L groups were higher than that of the burn + vehicle group. By transforming the data to the area under the wound area–time curve (AUC), compared to the burn + vehicle group, animals receiving SJM-L and SJM-H treatment showed significant improvement in wound healing (Fig. 4c).

### Effect of SJM on the histopathology of burn injury

**Fig. 5:**
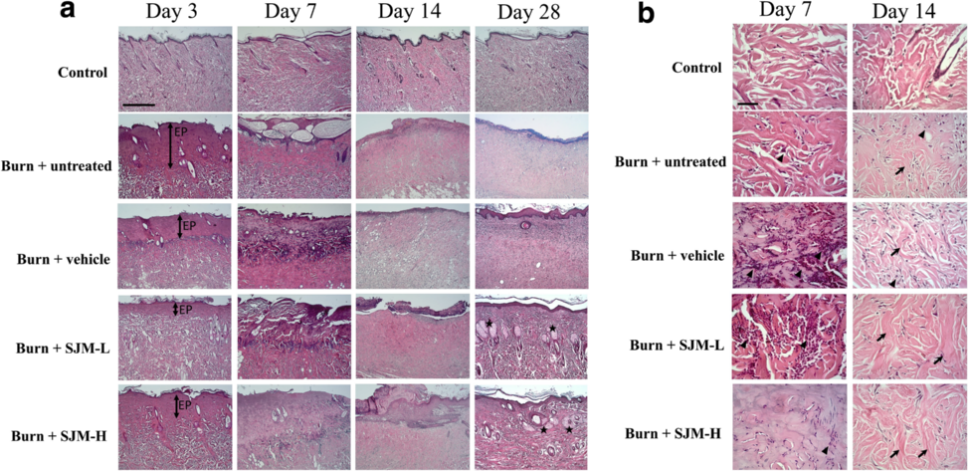
Histological evaluation of burn wound healing effect of SJM. **a** Paraffin wax tissue sections were stained with hematoxylin and erosin stain to observe the tissues of the burn wounds. Three days after the injury, hair follicle shedding and epidermis thickening were observed in each group, and the number of cells near the epidermis markedly decrased. After 7 days, the hair follicles of each group disappeared. Scarring was obervsed in the burn + SJM-L group, and the burn + SJM-H group exhibited collagen proliferation. After 14 days, the burn + untreated and burn + vehicle groups did not form the epidermis, whereas that of the burn + SJM-L group had begun to form, and a large amount of collagen was observed in this group. The burn + SJM-H group had already devleoped a complete epidermal layer along with a high deposition of collagen fibers. After 28 days, the burn + untreated group exhibited an incomplete epidermal structure, and granulation tissues were still observed. The burn + vehicle group had already grown a thicker epidermis compared with the injury group, but its hair follicles did not appear. The burn + SJM-L and burn + SJM-H groups not only exhibited complete epidermis but also large amounts of hair follicles, and their tissue fibers were neatly arranged. **b** Observing the magnified image of the corium layer revealed that after 7 and 14 days, the SJM-treated groups exhibited reduced neutrophil infiltration. Scales for (**a**) and (**b**) are 500 μm and 50 μm, respectively. EP, epithelial layer. ★ indicates hair follicle; ▲ indicates neutrophil infiltration; arrow indicates collagen fiber

Histopatholgical staining analysis was applied to examine the effect of SJM on wound tissues. After 3, 7, 14, and 28 days of burn treatment, the rats were sacrificed to obtain their skin samples, which were then stained using hematoxylin and erosin (Fig. 5a). Observing these images revealed that 3 days after injury, noticeable hair follicle shedding and epidermis thickening were observed in the burn + untreated, burn + vehicle, burn + SJM-H, and burn + SJM-L group, the number of cells near the epidermis was considerably reduced. Seven days after injury, the fair follicles of each group mostly disappeared, but the stratum corneum did not shed. The dermal collagen fibers were fused; hence, the shapes of the collagen fibers were not observable. In the dermis of the vehicle group, neutrophil infiltration was observed, indicating that the wound was still inflamed (Fig. 5b). Epidermal curst was observed in the burn + SJM-L group, the epidermis of which noticeably thickened, and the proliferation of collagen fibers was also observed. The burn + SJM-H group also exhibited proliferation of collagen fibers and a noticeable decrease in neutrophil infiltration. Fourteen days after injury, the epidermal layers of the injury and vehicle groups had not formed yet, and the hair follicles did not appear. Moreover, macrophage and neutrophil infiltrations were still observed in these two groups. By contrast, the epidermis of the burn + SJM-L group had begun to form, and a large amount of collagen fibers appeared. The burn + SJM-H group formed a complete epidermal layer, and a large amount of fibroblasts appeared. Twenty-eight days after injury, the burn + untreated group still did not form a complete epidermal structure, and granulation tissues were still observable. The burn + vehicle group formed a thick, uneven epidermal layer, but hair follicles did not appear yet. The burn + SJM-L and burn + SJM-H groups not only formed complete epidermal layers but also high amounts of complete hair follicles. Similar to those of the control group, the fibers of these groups were thick and neatly arranged, and no inflamed cells were observed. The aforementioned results revealed that after burn injury, although the wounds of the burn + untreated and burn + vehicle groups gradually decreased, the results of the tissue staining analysis revealed that the wounds were still inflamed, and an extensive period was required to form new epidermal layers and hair follicles. By contrast, the burn + SJM groups had begun to form epidermal layers after 14 days, and the granulation tissues developed more quickly and completely compared with those of the burn + untreated and burn + vehicle groups. After 28 days, the epidermal layers and hair follicles of the burn + SJM groups completely developed. Overall, SJM facilitates healing burnt tissues.

### Effect of SJM on increasing collagen and TGF-β1 contents in burn injury

**Fig. 6:**
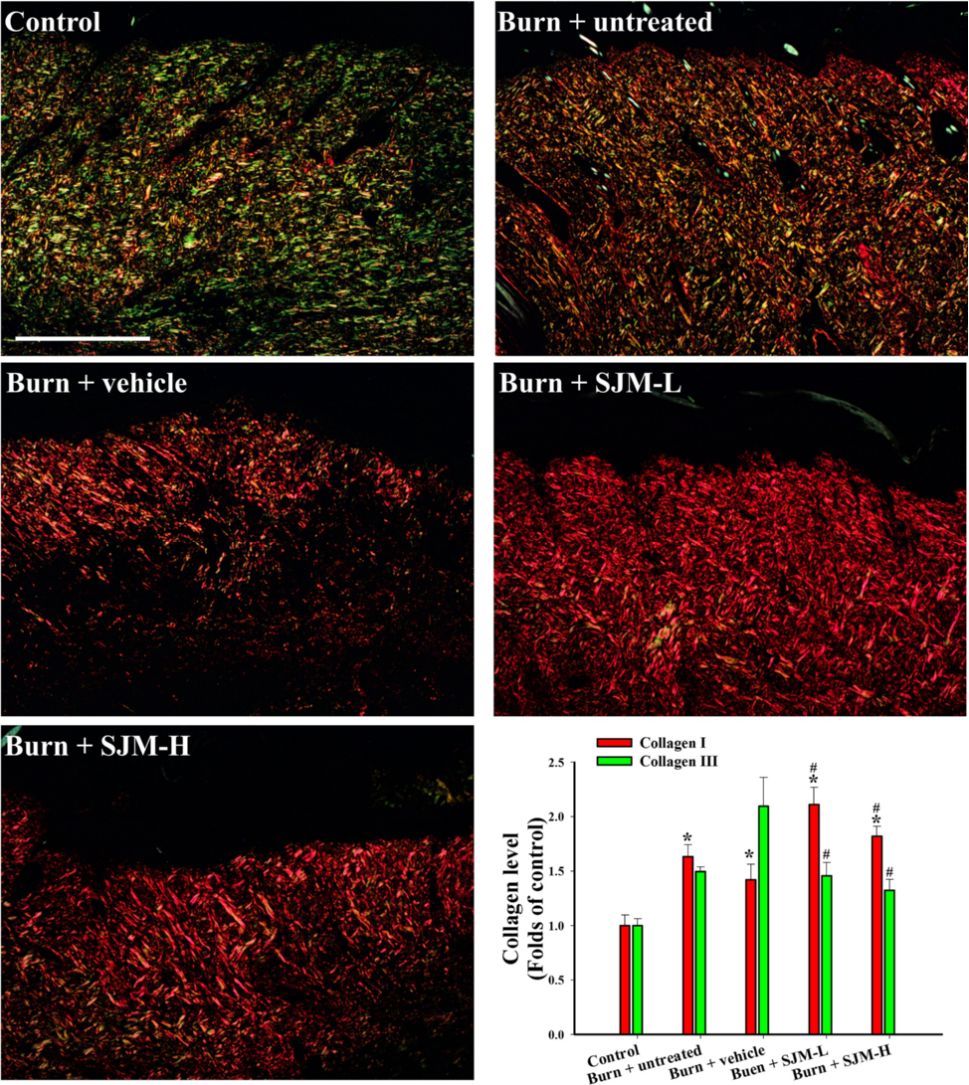
Determining the collagen contents of the SJM and vehicle groups using sirius red stain analysis. The paraffin wax tissue sections were stained with sirius red; hence, under a polarized light, collagen I and III appeared red and green, respectively. Fourteen days after burn injury, the collagen I content of each group significantly increased, with the burn + SJM groups having a higher content than did the burn + vehicle group. Conversely, the collagen III contents of the burn + SJM groups were significantly less than that of the burn + vehicle group. Scale = 500 μm; * denotes *p* < 0.05 compared with the control group and # denotes *p* < 0.05 compared with the burn + vehicle group

**Fig. 7:**
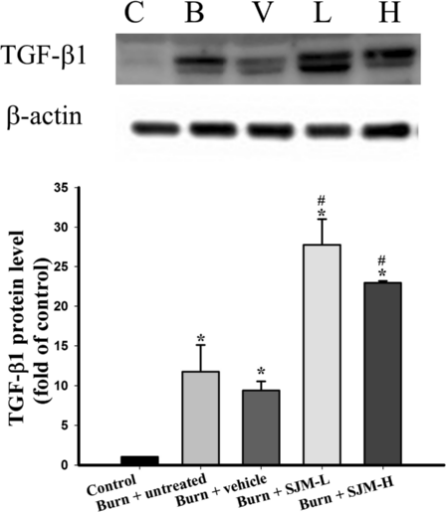
Effect of SJM on the TGF-β1 expression in the rats. After 7 days, the rats were sacrificed to collect their skin samples, and western blotting was used to examine the expression of TGF-β1. The results revealed that SJM enhanced the burn-induced upregulation of TGF-β1. * denotes *p* < 0.05 compared with the control group and # denotes *p* < 0.05 compared with the burn + vehicle group

Previous studies have indicated that the collagen content of a wound affects the proliferation and migration of the epidermal cells as well as the robustness of the wound. Therefore, the present study applied picrosirius red staining and a polarized light microscope, under which collagen I and collagen III respectively appear red and green. Next, ImageJ was used to quantified the red and green pixels in the images of the wounds (Fig. 6). The results revealed that after 14 days, the collagen I content of each group significantly increased, and the burn + SJM groups exhibited significantly greater collagen I deposition than did the burn + vehicle group. Conversely, the collagen III content of the burn + SJM groups were significantly less than that of the burn + vehicle group. To further analyze the potential mechanisms through which SJM facilitates healing wounds and increasing collagen content, we collected skin samples on the seventh day after burn injury, and western blotting was applied to investigate the TGF-β1 content (Fig. **7**). The results revealed that on the seventh day, the TGF-β1 content of the burn + SJM groups significantly increased compared with that of the control group. Compared with the burn + untreated and burn + vehicle groups, the burn + SJM groups demonstrated significantly increased TGF-β1 content.

## Discussion

This study applied in vitro and in vivo models to investigate the effect of SJM on wound healing. The results revealed that in the in vitro model, the presence of SJM decreased the iNOS expression and enhanced the functions of macrophages. The results of the rat burn injury model revealed that SJM decreased neutrophil infiltration, promoted wound healing, thus increasing the collagen I content and promoting the expression of TGF-β1 protein.

The healing process of a burn injury is generally divided into three sequential but interrelated phases, namely the inflammation phase, tissue formation phase (proliferative phase), and tissue remodeling phase [31]. Immediately following a burn injury is the inflammation phase, during which the injured blood vessels constrict within few minutes, and thrombocytes gather at the cortices of the injured blood vessels to prevent blood loss [32]. The injured area exhibits typical inflammation symptoms: redness, swelling, hotness, and pain. During the inflammation phase, thrombocytes secrete growth factors that signal leukocytes to enter the injured area. Neutrophils are the first to enter the injured area, followed by monocytes and lymphocytes [33]. Monocytes mainly function as the antibiotic mechanism of an organism and secrets a protease to remove denatured extracellular matrices. In the blood clots, thrombocytes secretes TGF-β, transforming monocytes in the injured area into macrophages, which remove cellular residuals in the injured area and secrete various types of cytokine including fibroblast growth factors (FGFs), TGF-β, platelet-derived growth factors (PDGFs), and epidermal growth factors (EGFs; [34]**)**. These cytokines also trigger the mechanism of granulation tissue formation.

Inflammation reactions induced by a burn injury can prevent infections caused by the invasions of foreign microorganisms. However, inflammation also damages nearby tissues [35]. Therefore, antiinflammation is also essential to wound treatment. Although the inflammation phase does not account for the majority of the overall wound healing process, previous studies have verified that cell activations and cytokine secretions during this phase can affect the final wound condition [36, 37]. The in vitro model of the present study revealed that SJM decreased the expression of iNOS induced by LPS. Moreover, observing the Day-7 and Day-14 tissue sections acquired from the animal model showed that compared with the burn + vehicle group, the burn + SJM groups exhibited decreased amounts of inflamed cells, indicating that SJM alleviates inflammations during wound healing. In the inflammation phase, macrophages mainly function to remove neutrophils. Specifically, macrophages first induce the apoptosis of neutrophils, which are then removed through phagocytosis [38, 39] to prevent inflammation from spreading. Afterward, some macrophages also undergo apoptosis, and the remaining macrophages continue to remain in the injured area and affect subsequent wound healing processes including collagen formation, angiogenesis, and reepithelialization [40]. The in vitro experiment revealed that SJM enhanced the phagocytic capability of macrophages; therefore, this study infers that the antiinflammation mechanism of SJM is related to macrophage function and reduced iNOS expression.

After the inflammation phase follows the proliferative phase, during which cells secrete cytokines that are then used by newly formed skins. Reepithelialization and angiogenesis are the most essential processes in the proliferative phase. During the process of wound healing, keratinocytes, fibroblasts, thrombocytes, and macrophages secrete TGF-β1 [41–44]. This protein exhibits a wide range of effects, influencing all types of cells involved in a wound healing process. Moreover, TGF-β1 is crucial to reepithelialization [45]. Mirza et al. [14] specifically ablate macrophages during wound healing and confirmed that macrophage ablation drastically slows the rate of wound healing, further verifying that TGF-β1 secreted by macrophages is essential to wound healing. Furthermore, the in vitro and in vivo experiments verified that TGF-β1 enhances the migration of keratinocytes [46, 47] and secretion of vascular endothelia growth factors (VEGFs) to promote angiogenesis [48]. Angiogenesis is mainly promoted through the TGF-β1, FGFs, and VEGFs secreted by macrophages [9–11]. Previous studies have confirmed that drug-induced activation and proliferation of macrophages enhance reepithelialization and wound strength [49, 50]. Using western blotting to examine the skin samples on the seventh day of burn injury revealed that SJM promoted TGF-β1 expression. Therefore, the effectiveness of SJM in promoting wound healing and epidermis formation should be related to how it activates macrophages to secrete TGF-β1.

The tissue remodeling phase is the final phase of wound healing and mainly features the dynamic balance between collagen formation and lysis. Collagen III fibers, which were formed during the initial wound healing process, are replaced with collagen I fibers. Moreover, collagen fibers are arranged into a desirable shape. The process in which the arrangement of collagen fibers gradually increases the wound strength [51] is called scarring, which is promoted by TGF-β1 and PDGFs [52, 53]. Particularly, TGF-β1 inhibits metalloproteinases, namely MMP-1, MMP-3, and MMP-9, and promotes the biosynthesis of metalloproteinase tissue inhibitor TIMP-1, thus inhibiting collagen degradation [54, 55]. Therefore, increasing the content of TGF-β1 facilitates wound healing processes, particularly the formation of collagen. The present study verified that seven days after burn injury, SJM promoted the expression of TGF-β1. Moreover, the sirius red staining experiment revealed that SJM facilitates converting collagen III to collagen I.

On the basis of previous studies, the chemical constituents of the shell of *Haliotis diversicolor* comprised Ba, Co, Cr, Cu, Fe, Mg, Mn, Na, P, Pb, Si, Sr, Ti, V, Zn, Ca, and CaCO_3_ and glutamic acid, aspartic acid, alanine, serine, and glycine [56, 57]. However, these chemicals may not be the major compounds that contribute to wound healing. Additional studies are warranted to confirm the major compounds that are involved in wound healing.

## Conclusions

According to the aforementioned results, we speculate that the effect and mechanism of SJM on promoting wound healing is related to macrophage activation. In the inflammation phase, SJM alleviates inflammation by inhibiting iNOS expression and removing neutrophils through phagocytosis. Furthermore, SJM induces the secretion of TGF-β1, converting collagen during the tissue remodeling phase. This study was the first to apply an evidence-based method to verify that SJM alleviates inflammation, enhances phagocytosis, and triggers wound healing. Moreover, SJM activates macrophages, which may promoting the secretion of growth factors. Subsequent studies can further examine whether SJM promotes growth factors.

## Abbreviations

AUC: Area–time curve
DMEM: Dulbecco’s modified Eagle’s medium
EGF: Epidermal growth factor
FGF: Fibroblast growth factor
iNOS: Inducible nitric oxide synthase
LPS: Lipopolysaccharide
MMP: Metalloproteinases
PBS: Phosphate buffer saline
PDGF: Platelet-derived growth factor
SJM: Shijueming
TGF**-**β1: Transforming growth factor-beta 1
TTBS: Tris-buffered saline
VEGF: Vascular endothelia growth factor
